# Enhancing communication in healthcare: Supports for interactions between adults with hearing loss and healthcare providers

**DOI:** 10.1371/journal.pone.0308592

**Published:** 2025-02-24

**Authors:** Ana Carla Garcia, Ali Arabi, Claire Croteau, Adriana Bender Moreira de Lacerda

**Affiliations:** 1 School of Speech-Language Pathology and Audiology, University of Montreal, Montreal, Quebec, Canada; 2 Research Center of the Institute universitaire de gériatrie de Montréal, Montreal, Quebec, Canada; Kasturba Medical College Mangalore / Manipal Academy of Higher Education, INDIA

## Abstract

**Background:**

Communication between a healthcare provider and an adult with hearing loss can be challenging and potentially contribute to health outcomes. To overcome this challenge, researchers have called for urgent multidisciplinary action on communication support in hearing healthcare.

**Objective:**

This scoping review examines the existing literature on communication support that can be provided to adults with hearing loss during their interactions with healthcare professionals.

**Methods:**

An online search was conducted using EMBASE, MEDLINE, CINAHL, and PsycINFO. All studies were imported into the Covidence platform for screening. The studies included in this analysis were published in English between 2013 and 2024.

**Results:**

Twelve articles were included in this review, and seven themes were identified: supportive nonverbal strategies, supportive verbal strategies, human support, environmental and physical adaptations, technological support, education and training, and psychological support and counseling.

**Conclusions:**

When communicating with a healthcare provider, adults with hearing loss can be assisted through supportive communication strategies.

## Introduction

Hearing loss is a sensory impairment, a hidden disability that affects many adults worldwide [1–4]. The World Health Organization (WHO) estimates that by 2050, hearing loss will affect about 10 percent of global population [5]. Hearing loss can lead to communication breakdowns when speech comprehension is impaired [5,6]. The miscomprehension of spoken messages creates barriers to efficient communication for adults with hearing loss (AwHL) and disrupts appropriate patient-clinician interactions [7–9]. Furthermore, AwHL may struggle to understand health-related information due to communication failure or misunderstanding verbal messages, which can result in poorer health outcomes compared to those without hearing loss [10,11].

Lack of support for communication can also negatively affect AwHL’s clinical outcomes and social interactions [10,12]. Communication failure in the clinical setting continues to adversely impact the well-being of people with hearing loss. Consequently, these individuals often experience a sense of exclusion, isolation, and frustration resulting from the absence of supportive strategies and adequate modifications to accommodate their communication requirements [13]. To prevent communication breakdown and sustain effective interactions, healthcare providers (HCPs) are advised to use supportive communication strategies for patients with hearing problems [6]. This support encompasses any strategy or adaptation that enhances communication and ultimately improves interaction [13,14].

Indeed, HCPs play a significant role in the health outcomes of patients with hearing loss [15]. When an HCP communicates effectively with an AwHL, the likelihood of errors in diagnosis or medication prescription is often reduced, leading to better patient outcomes [16,17]. Many individuals with hearing loss experience communication challenges during clinical visits to physicians or primary care providers [17]. To address this issue, it is necessary to conduct a study and update information about communication supports available to HCPs to improve interaction with AwHL.

## Methods

This scoping review was based on Arksey and O’Malley’s methodological framework [18] and Levac et al.’s recommendations [19]. The framework consists of five steps: 1. Identifying the research question; 2. Identifying relevant studies; 3. Selecting studies, 4. Charting the data, 5. Collating, summarizing, and reporting results. This study reviewed the existing literature on communication supports that can be used to improve interactions between HCPs and AwHL.

### Identifying the research question

Based on the current literature, the review was guided by the research question, “What communication supports can be used by HCPs to facilitate interaction with AwHL?”. The target population is comprised of HCPs who communicate with AwHL.

### Identifying relevant studies

This review focuses on scientific literature published between January 2013 and March 2024. The search strategy included keywords associated with the research question: 1. Communication support (i.e., communication strategies, supportive communication, communication techniques, communication facilitation, verbal/nonverbal strategies); 2. Adults with hearing loss (i.e., hearing impairment, adult hearing loss); and 3. Healthcare providers (i.e., healthcare providers, health professionals). A comprehensive search was performed using the following keywords across four electronic databases: EMBASE, CINAHL, MEDLINE, and PsycINFO. These databases were selected due to their extensive coverage of peer-reviewed journals relevant to the topic. The titles and abstracts of the studies were searched using keywords, and medical subject headings (MeSH) were employed whenever appropriate. A manual keyword search was also performed using Google Scholar.

### Study selection

**Eligibility criteria:** The study focused on HCPs and AwHL interacting in a clinical setting. The concepts examined involved communication supports and strategies employed or recommended to HCPs when communicating with AwHL. Observational and experimental studies using qualitative or quantitative methods were eligible for inclusion. Literature, such as dissertations, conference papers, and review studies, was not included. The inclusion criteria focused on HCPs interacting with AwHL and included studies published in English within the last 10 years. To be eligible, papers had to address the communication supports and strategies used with AwHL in healthcare settings. The communication supports/strategies sought were those assisting communication between HCPs and AwHL. Systematic, literature, and scoping reviews, opinion pieces, studies involving children, non-English studies, conference abstracts, informal publications, and studies where the HCPs had hearing loss were excluded

**Fig 1:**
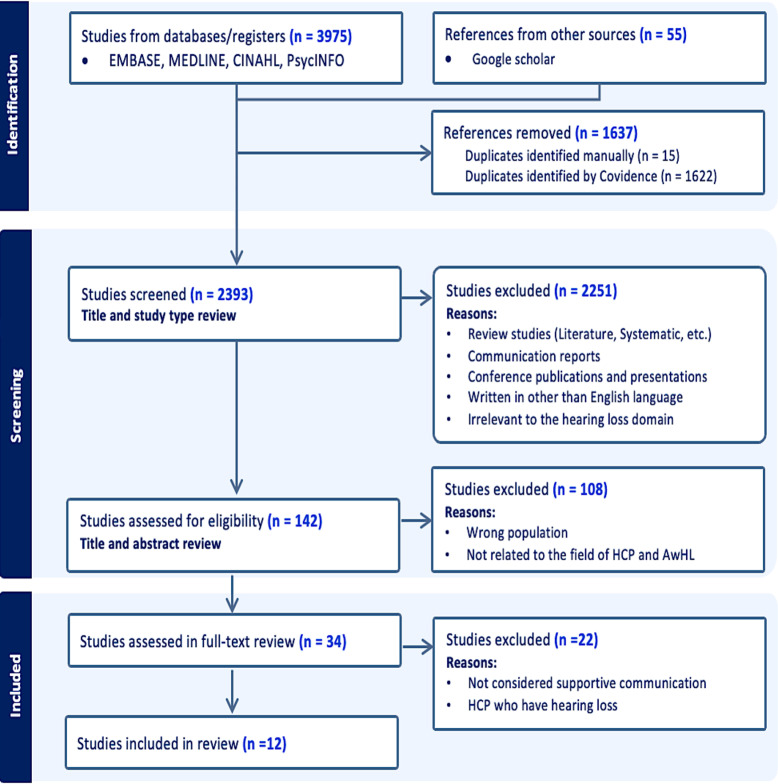
The data screening process.

**Study selection process and data collection:** All relevant studies were imported to the Covidence platform (https://www.covidence.org). Duplicate studies were removed, and the remaining studies were screened based on the inclusion criteria. Two reviewers (the first and second authors) independently assessed each study. Conflicting studies were discussed with the fourth author to arrive at a final decision. Full-text screening was conducted on eligible records, and a summary table was created to organize the extracted data. The first author completed the data charting with verification from the second and third authors to identify supportive communication strategies in each study. The data collection process, from identification to screening and inclusion, is illustrated in Fig 1.

### Charting the data

Two tables were created to chart the data. The first table included the studies’ characteristics, including descriptions of participants, objectives, study design, setting, and outcomes. The second table was used to organize the data related to communication supports extracted from the studies. A figure was added to consolidate recommended strategies for professionals.

## Results

The initial search yielded 4,030 studies. After removing duplicates, the first and second authors screened 2,393 records based on their titles and abstracts. Of these, 2,251 records were found to be ineligible and excluded. The remaining 34 articles were thoroughly evaluated in a full-text review, and 12 studies were included in the research sample. A PRISMA diagrammatic representation of the screening process is shown in Fig 1.

### Characteristics of the included studies

The participants in the included studies comprised diverse samples of HCPs (Table 1). The data originated from nine countries, with the USA being the most represented. Other studies presented data from multiple countries: Brazil, Italy, Japan, South Africa, Colombia, and Saudi Arabia. The data also included diverse groups of HCPs. The major participant categories were nurses (including registered nurses, nurse assistants, and nurse technicians), emergency medical service workers (including practitioners and students), and audiologists. Furthermore, community health workers were among the HCP population in the included studies. In the studies, HCPs, comprising physicians, physiotherapists, nutritionists, speech-language pathologists, dentists, pharmacists, and psychologists, were included as participants.

**Table 1:** Characteristics of the included studies and participant descriptions.

Study	Authors	Participant description	Objective	Design	Setting	Outcomes
1	Al-Wathinani et al., 2023 [20]	n = 57, Emergency medical services (EMS) students	To evaluate the knowledge of EMS students before and after an online knowledge transfer workshop on interaction with patients with HL and communication disorders.	Quasi-experimental/ Descriptive study	University	The online workshop was found to help enhance the level of communication knowledge among EMS students who interact with patients with HL and communication disabilities.
2	Rotoli et al., 2022 [21]	n = 148, EMS practitioners	To investigate EMS practitioners’ comfort levels when caring for individuals with hearing loss and identify current prehospital strategies and tools used by EMS practitioners to communicate with American Sign Language users.	Descriptive study	Emergency medical services	Many EMS practitioners reported finding it challenging to communicate with individuals who have HL and acknowledged that patients with HL often feel frustrated due to communication barriers. However, they found the educational training valuable and relevant to their clinical practice.
3	Ayala-Hernandez et al., 2021 [22]	n = 26, Deaf persons n = 6, HCP (medicine, physiotherapy, nursing, nutrition, psychology, and dentistry) n = 4, Interpreters	To promote an empathetic approach between HCPs and persons with HL, consider their contributions, and identify factors that facilitate the development of strategies to enhance healthcare services for these individuals.	Qualitative study	Association of the Deaf and university	Communication barriers exist between HL patients and HCPs, which prevent the development of interpersonal relationships and hinder the provision of adequate healthcare. HL patients must have access to an interpreter during healthcare processes to facilitate communication and improve healthcare services.
4	Harris et al., 2020 [23]	n = 3, AwHL (Deaf women)	Provide guidance on consulting with Deaf people in a neurology clinic.	Case study	Neurology clinic	By following some simple communication guidelines, such as adjusting to changes in the environment, the anxiety can be reduced not only for Deaf patients but also for clinical staff. This can enhance therapeutic communication, provide a more positive experience, and increase the chances of a beneficial patient outcome.
5	Stevens et al., 2019 [24]	n = 1581, AwHL	To better understand the experiences of individuals with HL in healthcare settings and provide recommendations for improvement of outcomes and access by identifying unmet needs and communication barriers.	Cross-sectional/Descriptive study	Primary care	The study highlights the difficulties experienced by individuals with hearing loss, particularly older adults, when navigating primary care visits and healthcare providers.
6	Ruesch, 2018 [25]	n = 339, Registered nurses	To create a knowledge assessment tool that measures the knowledge of registered nurses regarding hearing impairment, hearing aids, assistive devices, communication strategies with HL patients, and policies related to caring for persons with HL.	Descriptive study	Nursing units of hospital	There are knowledge gaps related to HL and effective communication strategies when caring for HL patients, highlighting a need for an educational intervention to better equip registered nurses with the necessary information on all aspects of hearing impairment and effective communication strategies when caring for HL patients.
7	Orrie & Motsohi, 2018 [26]	n = NA, Healthcare workers	To better understand the challenges HCPs face in managing patients with hearing impairment in a primary healthcare setting.	Qualitative/Descriptive Case study	Community health center	HCPs face challenges in serving Deaf clients due to language barriers. Despite difficulties in communication, healthcare providers believe these barriers can be overcome.
8	Marquete VF, Costa MAR, Teston EF., 2018 [27]	n = 198, Nurses, nurse technicians, and nursing assistants	To investigate the extent of knowledge and training among HCPs for effective communication with individuals with HL.	Cross-sectional	Public health system	HCPs found that communication barriers with hearing-impaired individuals are often due to a lack of knowledge on how to communicate. The study showed that this lack of knowledge is due to the absence of training opportunities, often caused by factors such as a shortage of time and financial resources.
9	Pendergrass et al., 2017 [28]	n = 10, Nurse practitioners	To investigate the perspectives of nurse practitioners regarding the challenges and opportunities that arise when providing healthcare services to deaf individuals who use American Sign Language	Descriptive/Qualitative study	Primary medical and mental health care	Nurse practitioners preferred the use of interpreters to support their visits, but they were not aware of the significance of their role in ensuring effective communication. A professional sign language interpreter was viewed as a last resort when other communication methods had proven unsuccessful.
10	Sánchez et al., 2017 [29]	n = 12, Community health workers	To evaluate the practicality of educating community HCPs about HL as a potential means of expanding the availability of hearing health support services in an underprivileged area, with the goal of recognizing HL indications among community members and employing effective communication strategies and assistive devices.	Cross-sectional	Community health service	The extensive training proved successful in enhancing HCPs’ understanding of hearing loss and audiology-related ideas, and in boosting the community health workers’ assurance to facilitate a support group for individuals with hearing loss.
11	Ekberg et al., 2017 [30]	n = 26, Audiologists n = 63, AwHL	To investigate the situations in which clients use repair during their initial audiology appointments and to examine the context of interaction in which these requests occur.	Cross-sectional	Audiology clinic	Audiologists employ various strategies during their responsive turn to effectively address communication disruptions. This reinforces the significance of communication, particularly for older AwHL and emphasizes the role of mutual gaze in fostering effective communication.
12	Hyoguchi et al., 2016 [31]	n = 20, Deaf n = 19, Hard of Hearing n = 20, Hearing	To assess the understanding and attitude of deaf participants to medication information provided by pharmacists and determine the effectiveness of medication education lectures in improving their knowledge and confidence in medication use.	Cross-sectional/ Descriptive study	Association of hearing-impaired people	Deaf participants had a lower understanding of medication use than hearing participants before the medication education lecture, and adjusting medication information provided by pharmacists according to the recipient’s reading level could potentially improve deaf patients’ knowledge, but it may not be sufficient to increase their comprehension levels significantly.

* AwHL=Adult with hearing loss, HCPs = Healthcare providers, HL=Hearing loss, EMS = Emergency medical service.

Many studies (n = 7) were conducted in public health systems, such as nursing units of hospitals, primary medical health care, community health workers, and emergency medical services. The other settings were private clinics (n = 2), associations with hearing-impaired people (n = 2), and universities (n = 2). Seven of the studies were descriptive, three were qualitative, and two were in mixed methods.

### Identified supportive strategies

The seven key supportive communication strategies identified are summarized in Table 2 and can be employed by HCPs when interacting with AwHL. The strategies used were: 1. nonverbal supportive strategies; 2. verbal supportive strategies; 3. human support; 4. environmental and physical support; 5. technological support; 6. education and training; and 7. psychological support and counseling.

**Table 2:** Communication supports identified from the included studies.

Communication support	Number of studies	Examples
Supportive non-verbal strategies [20–24,26,27,30,31]	9	Use mimicry, facial expressions, mutual gaze, and eye contact Communicate through writing, drawing, reading, and written notes Have face-to-face communication and look at the patient’s face Use hand signals, physical gestures, and body language Provide enough time for the patient to understand and explain Allow the patient to lip read Use cards, pictures, and videos to aid communication Illustrate procedures on screen
Supportive verbal strategies [21,23,24,30,31]	5	Speak clearly at a normal volume and slow pace Use simple words, short sentences, and keywords Use repetition and rephrase words and phrases Avoid interrupting the patient when they are speaking Use understandable medical terminology Adapt communication content to the level of the patient Avoid abstract expressions, double negatives, and technical words Ask patients for opinions, reassurance, and permission Use stories or clear examples to explain symptoms Use closed-ended or open-ended questions based on the context
Human support [21,22,26–28]	5	Use sign language interpreters or designated healthcare translators Ask family members for translation and interpretation Help create a network of community-based interpreters
Environmental and physical adaptations [23,24,26,29]	4	Use visual demonstrations and signs Use noise dampening materials Use alerting devices in the waiting room and adjust lighting and space Adjust position and proximity to the patient Use tactile modifications
Technological support [22,24,29]	3	Use adaptive hearing devices Use amplified phones, FM systems, and microphones Use text messaging and email instead of phones Use the speakerphone function or a two-way headset
Education and training [20,25,29]	3	Participate in virtual knowledge transfer workshops related to HL P articipate in short-term educational courses related to hearing aids Train and sensitize colleagues and partners to the patient’s status
Psychological support and counseling [23,29]	2	Consult psychosocial issues in the diagnosis and management phases Consult communication breakdowns Show empathy, agreement, and disagreements with patient Identify negative emotions that arise due to HL

## Discussion

**Fig 2:**
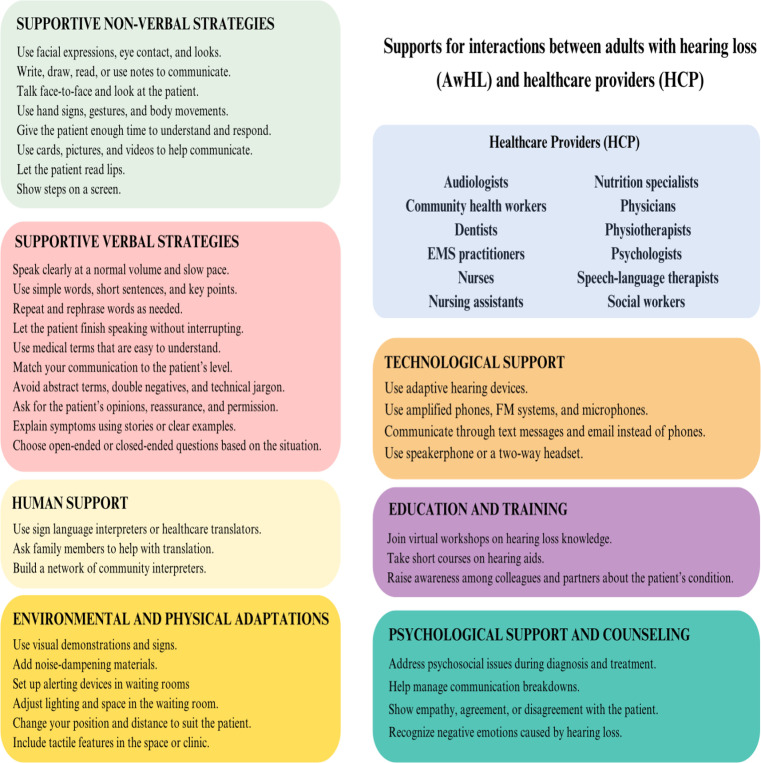
Recommended communication supports and targeted HCP identified in included studies.

The objective of this scoping review was to understand the communication supports that can be employed by HCPs to enrich interactions with AwHL. Health professionals can use these supports in different contexts (Table 1) to optimize communication in clinical practice. Seven concepts were identified (Table 2, Fig 2).

According to the current literature, supportive nonverbal strategies were mostly used by HCPs to enhance interactions with AwHL [20–24,26,27,30,31]. Using gestures such as arm movements, hand movements, mutual gaze, eye contact, and writing has been reported as a supportive nonverbal communication strategy to facilitate the access of patients with hearing loss to the healthcare system [21,23,30]. Many emergency medical services recommend using hand signals, physical gestures, and body language [16,17,20,23]. People with hearing loss rely heavily on visual cues to understand speech, particularly under adverse hearing conditions [32]. Hyoguchi et al. (2016) recommended that pharmacists use nonverbal strategies to provide AwHL with necessary information about prescribed medicine. These strategies include gestures, facial expressions, and printed slides [31]. Individuals with hearing loss typically comprehend messages more effectively when they can both see and listen to the speaker, as opposed to when they can only hear the message. Such an audiovisual advantage can convey extra information, leading to better speech comprehension, particularly under adverse hearing conditions [33].

Effective verbal communication is a critical component of information exchanges. When AwHL fail to understand the spoken information conveyed by HCPs, communication breakdown can occur. As such, HCPs must collaborate with their patients and employ communication support to overcome these challenges [6,34,35]. Community HCPs are highly encouraged to employ supportive verbal strategies, including slower speech rates, shorter phrases, appropriate volume, and avoidance of complex medical terminologies when interacting with patients with hearing loss [29,32]. In addition, HCPs are advised to provide educational lectures tailored to their AwHL literacy levels when explaining medication-related information [31]. Therefore, healthcare professionals must familiarize themselves with these verbal techniques and implement them to enhance professional-patient communication. Using verbal support enables healthcare professionals to address the identified gaps in effectively communicating with AwHL [6].

Human support for communication has been accentuated in many studies, among which sign language interpreters and assistance were emphasized and recommended for healthcare workers in medicine, physiotherapy, nursing, nutrition, psychology, dentistry, and primary and emergency medical services [21,22,26–28]. The American Disabilities Act (ADA, 1990) mandates that healthcare providers ensure equal healthcare treatment and effective communication for individuals who are deaf or hard of hearing [35]. Despite this directive, deaf and AwHL still face significant communication barriers in healthcare [22, 35]. These barriers can result in negative healthcare events due to a lack of clear communication between AwHL and HCPs [27]. In a primary medical and mental healthcare context, Pendergrass et al. (2017) investigated nurse practitioners’ perspectives on the challenges and opportunities that arise when providing services to deaf individuals. Their findings showed that nurse practitioners prefer to use interpreters to support their visits [28]. Moreover, if other communication methods fail, nursing practitioners should consider seeking official single-language interpreters [26]. Ayala-Hernandez et al. (2021) attempted to study  the communication barriers between HCPs (including medicines, physiotherapists, nurses, nutritionists, psychologists, and dentists) and patients with hearing loss. The report suggested that AwHL should be provided with interpreters during their visits to healthcare services [22].Therefore, HCPs can use human support strategies to better understand AwHL’s clinical symptoms during medical histories and make better treatment decisions.

Environmental and physical adaptations can support interaction and lead to more successful and effective communication in diverse healthcare settings [26,29]. Stevens et al. (2019) conducted a survey of 1,581 self-report individuals with hearing loss in primary care settings. The clinical implications of this study necessitated adjustments aimed at improving communication, particularly in enhancing the interaction between community HCPs and patients [24]. This aim was achieved by ensuring appropriate lighting, clear speech, and implementing environmental modifications, such as using tactile or visual aids in waiting areas, installing noise-absorbing materials (carpeting and curtains), and avoiding the use of telephones for conveying important health information [24]. Other studies have indicated that the environment should be designed to accommodate individuals with hearing loss, including seating them near the receptionist, utilizing heavy draping or carpeting to minimize echoes, and employing visual cues in clinics and public health systems [23,26,29]. Stans et al. (2017) assert that healthcare professionals should be aware of the potential influence of environmental elements on conversations, because small adjustments to the physical environment have implications for rehabilitation and can contribute to a communication-friendly environment for conversations with people who find verbal communication challenging [36].

AwHL have the highest risk of experiencing social isolation, and using hearing aids may help overcome the loss of signal intensity and optimize speech intelligibility to maintain good interactions. In the healthcare context, the utility of technological support for AwHL can be accomplished through text messaging and email instead of phone calls, as well as employing the speakerphone function or a two-way headset [22,24,29,37].

Education and training in the field of hearing loss have been recommended to community HCPs, audiologists, nurses. This aim could be achieved by attending virtual knowledge transfer workshops focusing on hearing loss and short-term educational courses covering the basics of hearing aids [20,25,29]. Marquete et al. (2018) investigated the extent of knowledge and training among nurses, nurse technicians, and nursing assistants regarding effective communication with individuals with hearing loss. The authors found that communication barriers with deaf individuals are often caused by HCPs’ lack of knowledge of how to communicate, and that this is due to the absence of training opportunities [27]. Accordingly, it has been recommended that registered nurses and emergency medical service workers attend communication training courses focused on hearing loss, which can take the form of online knowledge transfer workshops or short-term training programs [21,25].

Psychological support and counseling can be used by HCPs to address AwHL’s psychosocial issues [23,29]. It is recommended that HCPs consult with patients about the communication breakdowns they experience due to hearing loss, show empathy, and discuss their agreements and disagreements with patients while identifying negative emotions that arise due to hearing loss. Studies with community health workers, audiologists, and neurologists reveal that an absence of communication support in the workplace has a detrimental effect on the well-being of individuals with hearing loss, resulting in feelings of isolation and frustration [23,29]. To better support individuals with hearing loss in healthcare systems, it is crucial to develop psychological support programs that consider the specific needs of this population [38].

This scoping review contributes to an understanding of supportive communication strategies for healthcare providers in hearing healthcare and makes recommendations for optimizing communication in specific care situations. Any profession can use communication support, but its use will depend on the context of each professional. Future research could investigate the broader impact of combining verbal and nonverbal communication strategies. It could also examine whether using multiple supportive communication strategies simultaneously yields better outcomes in long-term care facilities and hospitals than using single strategies. Using this study’s results, we developed an evidence-based brochure by gathering information from current research, which can be shared with healthcare providers in hospitals, rehabilitation centers, clinics, pharmacies, and other relevant institutions associated with the Institut Universitaire de Gériatrie de Montréal (IUGM).

## Limitations and strengths

This scoping review had some limitations. Studies published in languages other than English with no accompanying translation were excluded. Language bias was present in this review because most of the included studies were in English and were performed with convenience samples from health professionals and/or patients, which may not be representative of all communication supports used by people with hearing loss.

## Conclusion

Healthcare professionals can utilize various communication supports to interact with adults who experience hearing loss. These supports include nonverbal, verbal, human, environmental and physical, technological, education and training, and psychological support and counseling. The choice of communication supports will depend on the context (place and situation) in which each professional finds themselves. According to the studies included in this scoping review, the most frequently utilized concepts were nonverbal, verbal, and human support.

## Supporting information

S1 Table Scoping review checklist.
(DOCX)
